# Permissive hypotension does not reduce regional organ perfusion compared to normotensive resuscitation: animal study with fluorescent microspheres

**DOI:** 10.1186/1749-7922-7-S1-S9

**Published:** 2012-08-22

**Authors:** Bruno M Schmidt, Joao B Rezende-Neto, Marcus V Andrade, Philippe C Winter, Mario G Carvalho, Thiago A Lisboa, Sandro B Rizoli, Jose Renan Cunha-Melo

**Affiliations:** 1 Federal University of Minas Gerais, Av. Prof. Alfredo Balena 190, Belo Horizonte, MG, 30130-100, Brazil; 2 Departments of Surgery and Critical Care Medicine, Sunnybrook Health Sciences Centre, University of Toronto, Canada; 3 Rua Dr. Riggi 133 – Le Cottage, Nova Lima, MG, 34000-000, Brazil

## Abstract

**Introduction:**

The objective of this study was to investigate regional organ perfusion acutely following uncontrolled hemorrhage in an animal model that simulates a penetrating vascular injury and accounts for prehospital times in urban trauma. We set forth to determine if hypotensive resuscitation (permissive hypotension) would result in equivalent organ perfusion compared to normotensive resuscitation.

**Methods:**

Twenty four (n=24) male rats randomized to 4 groups: Sham, No Fluid (NF), Permissive Hypotension (PH) (60% of baseline mean arterial pressure - MAP), Normotensive Resuscitation (NBP). Uncontrolled hemorrhage caused by a standardised injury to the abdominal aorta; MAP was monitored continuously and lactated Ringer’s was infused. Fluorimeter readings of regional blood flow of the brain, heart, lung, kidney, liver, and bowel were obtained at baseline and 85 minutes after hemorrhage, as well as, cardiac output, lactic acid, and laboratory tests; intra-abdominal blood loss was assessed. Analysis of variance was used for comparison.

**Results:**

Intra-abdominal blood loss was higher in NBP group, as well as, lower hematocrit and hemoglobin levels. No statistical differences in perfusion of any organ between PH and NBP groups. No statistical difference in cardiac output between PH and NBP groups, as well as, in lactic acid levels between PH and NBP. NF group had significantly higher lactic acidosis and had significantly lower organ perfusion.

**Conclusions:**

Hypotensive resuscitation causes less intra-abdominal bleeding than normotensive resuscitation and concurrently maintains equivalent organ perfusion. No fluid resuscitation reduces intra-abdominal bleeding but also significantly reduces organ perfusion.

## Introduction

Severe hemorrhage is a major cause of death in the trauma patient. Approximately 45% of pre-hospital deaths and 55% of the deaths after hospital admission for trauma are caused by exsanguination [1]. Trauma related hemorrhage caused by penetrating torso injury is a quick killer [1,2]. A study of time to death from trauma showed that among those who died in the first 24 hours, 35% were pronounced dead within the first 15 minutes, thoracic vascular injuries from penetrating mechanisms were the main cause; deaths occurring within the first 16 to 60 minutes showed similar results [2]. Therefore, successful treatment of trauma related hemorrhagic shock should involve timely control of the bleeding and maintenance of adequate tissue perfusion, especially in penetrating mechanism [3].

The importance of fluid resuscitation to maintain tissue perfusion in hemorrhagic shock has been well established, but the optimal blood pressure capable of providing adequate organ perfusion without augmenting hemorrhage is currently a topic for research [3-9]. Recent clinical studies on permissive hypotension and damage control resuscitation aiming at delivering higher ratios of blood products and decreasing crystalloid infusion have led to fewer complications associated with excessive fluids, less coagulopathy and ultimately increased survival [6,7].

Several investigators demonstrated, in animal models, that permissive hypotension (PH) or hypotensive resuscitation (mean arterial pressure between 50-65 mmhg) resulted in decreased blood loss and ultimately lower mortality in hemorrhagic shock compared to normotensive resuscitation [10-14]. Our group recently demonstrated that enhanced clot formation is one of the mechanisms involved in the reduction of blood loss in hypotensive resuscitated animals [15]. However, conflicting results have been shown in other experimental studies including lower survival rates with hypotensive resuscitation [16,17]. Furthermore, concerns have been raised over inadequate fluid resuscitation with deleterious hemodynamic and organ perfusion effects [18,19]. Therefore, experimental models to study fluid resuscitation related to traumatic hemorrhage should be clinically relevant, and contemplate timing and sequence of events that take place in urban or military trauma [13,20]. Also important are research tools capable of providing information about the actual consequences of different resuscitation strategies on organ perfusion; one useful tool is the microsphere deposition method [21-24].

In a previous study with radioactive microspheres moderate volume resuscitation improved organ perfusion with less bleeding after venous hemorrhage compared to large volume or no volume [25]. In that study, the interventions were not designed to simulate a real trauma scenario, and the resuscitation regimen used was not pressure guided [25].

The objective of this study was to investigate regional organ perfusion acutely following uncontrolled hemorrhage in an animal model that simulates a penetrating vascular injury and accounts for prehospital times in urban trauma. We set forth to determine if hypotensive resuscitation (permissive hypotension) would result in equivalent organ perfusion compared to normotensive resuscitation.

## Materials and methods

The study was approved by the Animal Research and Ethics Committee of the Federal University of Minas Gerais, Belo Horizonte, Brazil, and conducted under stringent animal ethics protocol.

## Animals

Male Wistar rats (250-335 g) were housed in groups of 3 in appropriate cages, and maintained at 25^o^C on 12-hour light/dark cycles. Animals were acclimated for 2 weeks before the experiment, were fed rat chow (Purina® Ratochow, Caxias, RS, Brazil) and water *ad-libitum*.

## Monitoring procedures

Animals were anesthetized with 60 mg/kg of ketamine and 15 mg/kg of xylazine (Rhabifarma Industria Farmaceutica Ltda., Hortolandia, SP, Brazil) by intraperitoneal injection. Additional doses of ketamine and xylazine were administered intravenously, 2.5 mg/kg and 1mg/kg respectively. Operative sites were prepared with 10% povidone iodine solution. A tracheotomy was performed, and a segment of a 14 G intravenous catheter (Smiths Medical do Brasil, Sao Paulo, SP, Brazil), approximately 2.5 cm in length, was inserted into the trachea. The left internal jugular vein, the right carotid artery, and the right femoral artery were cannulated with polyethylene tubing (PE 50; Clay Adams, Sparks, MD) prefilled with heparinized saline solution (Parinex® Hipolabor, Sabara, MG, Brazil). Mean arterial blood pressure (MAP) and heart rate (HR) were continuously monitored with a Biopac (Biopac Systems Inc., Goleta, CA) connected to the right femoral artery after five minutes stabilisation period. The tubing in the right carotid artery was further advanced into the left ventricle, as evidenced by the typical left ventricle pressure curve (approximately 100 mmHg); left ventricular pressure was continuously monitored (Biopac Systems Inc., Goleta, CA).

## Microspheres injection

Fluorescent polystyrene microspheres (FluorSpheres®, Invitrogen Molecular Probe®, Eugene, OR), 15 μm in diameter, were suspended in solution (0.15 M of NaCl 0.05%, Tween 20, and 0.002% Thimerisol). Microspheres containing red fluorescent dyes (absorption/emission wavelength 580/605 nm), blue-green (505/515 nm), blue (625/645 nm), and orange (540/560 nm) were used. Microspheres were vortexed for one minute, followed by sonication, for one minute, to prevent flocculation. After sonication, 0.3 ml of the microsphere solution, approximately 300,000 microspheres, was aspirated into a 1ml syringe (Becton Dickinson Ind. Cir. Ltda., Curitiba, PR, Brazil). The right femoral artery catheter and the right carotid artery catheter were temporally disconnected from the monitor before injection. The carotid artery catheter was connected to the 1 ml syringe containing the microsphere solution of a chosen color. The right femoral artery catheter was connected to a peristaltic roller pump (Minipuls 3 Gilson, Villiers Le Bel, France) preset to remove blood at a rate of 0.7 ml/min into a test tube. Twelve seconds after the beginning of the removal of blood, 0.3 ml of the microsphere solution was injected into the carotid artery catheter over 20 seconds. Blood removal persisted for a total of 90 seconds. The carotid artery catheter was flushed with 2 ml of LR during the last 60 seconds of blood removal to prevent microspheres adhesion to the inner surface of the catheter and to replace the volume of blood removed.

## Experimental groups

Twenty four (n=24) animals were randomly divided (table of random numbers) into four groups (n=6 animals per group) according to the fluid resuscitation regimen used. Normal blood pressure group (NBP) underwent normotensive resuscitation with intravenous LR to maintain MAP at baseline (pre-hemorrhage) values. PH group received LR to maintain MAP at 60% of baseline. A third group received no resuscitation fluid (NF) after bleeding, and in a fourth group sham operated animals underwent pre-hemorrhage procedures but no bleeding.

## Hemorrhage procedures

**Figure 1:**
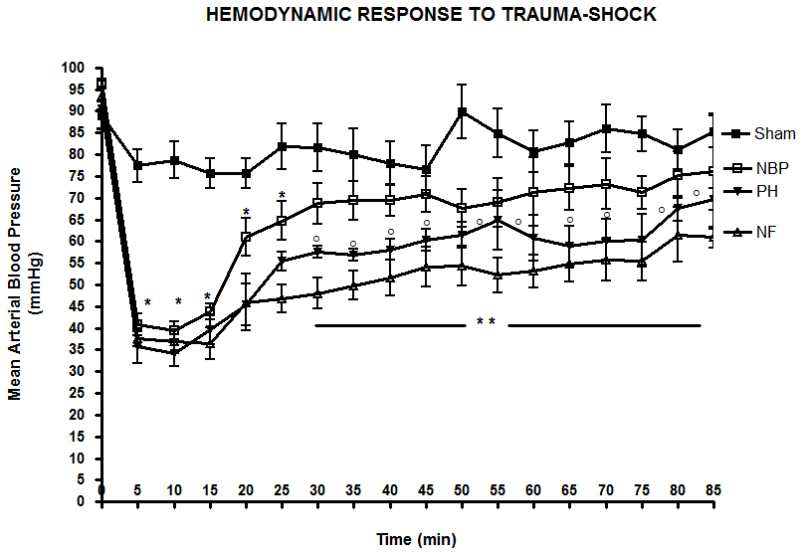
**Mean arterial blood pressure during resuscitation.** Lactated Ringer’s infusion was started at 15 minutes in the NBP and PH groups. Data represent mean ± SD (6 animals per group). * *p* < 0.05 NF, NBP and PH vs. Sham; ° *p* < 0.05 PH vs. Sham and NBP; ** *p* < 0.05 NF vs. all other groups; two-way analysis of variance (ANOVA). NF = No Fluid; NBP = Normal Blood Pressure; PH = Permissive Hypotension.

A midline laparotomy (4cm) was performed to expose the infra-renal aorta, and a 3-0 nylon (Polysuture®, Sao Sebastiao do Paraiso, MG, Brazil), continuous full thickness running suture, was placed through the edges of the laparotomy to close the abdomen immediately after the aortic injury. Bleeding was induced by a single puncture injury to the infra-renal aorta with a 25G needle (Becton Dickinson Ind. Cir. Ltda., Curitiba, PR, Brazil); time point one (T1). The abdomen was immediately closed by pulling on the previously placed sutures. For the next 15 minutes, no fluids were infused to simulate the average time that emergency medical services (EMS) take to arrive at the scene [13]. Only animals that had a drop in MAP to ≤ 40 mmHg, and were alive up to 15 minutes after the aortic injury were used in the study; otherwise, the animals were euthanised and replaced. After 15 minutes (T2) lactated Ringer’s solution (LR) (Fresenius Kabi®, Aquiraz, CE, Brazil) was continuously infused through tubing in the internal jugular vein (IJ) using a peristaltic roller pump (Minipuls 3 Gilson, Villiers Le Bel, France) for 70 minutes (T3 – end of the experiment) (Figure 1). Fluid infusion rate was adjusted to maintain MAP within the preset limits for each experimental group; maximum infusion rate was 1.4 ml/Kg/min. Blood samples for laboratory tests (hemoglobin (Hb), hematocrit (Hct), platelet count, and lactic acid were obtained at the end of the experiment (T3). The abdomen was then opened and total intra-abdominal blood loss was calculated as the difference between blood-soaked sponges minus the weight of preweighed dry sponges. Animals were euthanised with an anesthetic overdose at the end of the experiment.

## Fluorescent microsphere recovery

At the end of the experiment, but before blood sampling for laboratory tests and the intra-abdominal blood loss calculation, a microsphere solution of different color from the one used in the beginning of the experiment was injected in the right internal carotid artery catheter. Blood was withdrawn from the catheter in right femoral artery as previously described. The left cerebral hemisphere, heart, lungs, mesentery, pancreas, spleen, the right and the left kidneys were removed and individually weighed. Samples weighing 1.5 g were taken from the liver to determine hepatic arterial blood flow. The bowel and the stomach were opened longitudinally and washed with normal saline to remove gastrointestinal secretions before weighing. All organs were individually placed inside a centrifuge tube (35 mm x 105 mm) (Sorval Legend Mach 1.6-R, Thermo Scientific, Waltham, MA) with tissue digestion solution (2M KOH 44.8g + Tween 80 (0.5%) 8ml + 99% IMS ethanol (Vetec Quimica Fina Ltda, Rio de Janeiro, Brazil) for 6h in water bath. Tubes were then vortexed and sonicated until complete dissolution of the fragments, followed by centrifugation (1500 g for 15 minutes). The supernatant was carefully aspirated leaving the pellet with microspheres. To prevent microspheres flocculation, 1 ml of dH_2_O was added to the pellet which was briefly vortexed, followed by the addition of 9 ml of ethanol-Tween (100% ethanol + 0.5% Tween-80). The resuspended pellet of microspheres was centrifuged at 1500 g for 15 minutes, the supernatant carefully aspirated, 5 ml of 0.1 M phosphate buffer (pH 7.0), and 4 ml of absolute alcohol were added to the pellet and vortexed briefly before spinning at 1500g for 20 minutes. The supernatant was carefully aspirated and 4 ml of ethyl acetate-98% (Labsynth, Diadema, SP, Brazil) were added to the pellet and vortexed several times over 3-5 minutes. Tubes were kept in a dark room for 10 minutes to avoid photobleaching of the fluorescent dyes, and readings were performed within one hour with a spectrofluorometer (Shimadzu Scientific Instruments UV-3600-UV-VIS-NIR, Columbia, MD) preset to determine fluorescence within excitation and emission wavelengths of each color of microsphere used in the study.

## Calculation of organ blood flow

The deposition of microspheres in an organ is proportional to the fluorescence intensity. Therefore, to calculate the number of microspheres in a particular organ, the fluorescence in the organ is compared to that of commercially available preparations with a known number of microspheres; 10 μl of FL_10_ contains 10,000 microspheres (Sample Fluorescence (FS)/Sample Microspheres (MS) = FL_10_/10,000). To reduce experimental error a conversion factor (CF) was calculated as the average of the sum of the fluorescence of 2500 microspheres/ml in ethyl acetate-98% solution, as well as the fluorescence of 1250 microspheres/ml, and that of 625 microspheres/ml; 2 ml of each solution was used. The number of microspheres in each experimental sample was calculated by multiplying the CF obtained for the fluorescent dye used in the sample by the actual fluorescence of the sample (MS = FS x CF). Blood flow to an individual organ (*Q*) was calculated using the number of microspheres in the sample (MS), the number of microspheres in the reference blood sample (MRBS), the weight of the sample (W), and the reference flow (RF), as in the formula: *Q* = MS/MRBS x RF/W. To obtain the reference flow (RF) the density of blood (1.06 ml/g) is multiplied by the blood sample withdrawal speed (1.5 ml/min), and then divided by the weight of the reference blood sample. To determine portal blood flow to the liver, fragments weighing 1/5 of the weight of each organ that drained to the portal system were obtained and grouped as a single sample. The result of the blood flow (*Q*) in that sample was multiplied by five and then divided by the total weight (g) of the liver of the animal to obtain the portal blood flow per gram of liver parenchyma. Cardiac output (CO, ml/min) was calculated by taking the amount of microspheres injected in the left ventricle (MLV = 300,000) divided by the number of microspheres in the reference blood sample (MRBS) multiplied by the reference flow (RF), as in the formula: CO = MLV/MRBS x RF. Cardiac index (CI) was calculated using the formula: CI = CO x (W x 100)^-1^.

## Statistical analysis

Data are reported as the mean ± SD. One-way or two-way analysis of variance (ANOVA) was performed with post hoc analysis using Tukey’s test for multiple comparisons between e experimental means. To access interaction between variables the conditions NF, NBP, and PH were modeled in a factorial analysis of variance. The unpaired Student’s *t* test was used to analyse comparisons between two groups. A *p* < 0.05 was considered statistically significant.

## Results

Mean animal weights in each group were 304 ± 20.4 g (Sham), 298 ± 27 g (NF), 302 ± 22.0 g (NBP), and 292 ± 40 g (PH); (*p* > 0.05). The amount of anesthetics used was similar between the groups (ketamine 108.5 mg/Kg ± 10.2 to 122± 35 mg/Kg; xylazine 19.3 ± 3.6 mg/Kg to 20.5 ± 7.4 mg/Kg). The total mortality rate in the study was 34%, approximately 50% of the deaths occurred within the first 15 minutes after the aortic injury. There were no statistical differences in mortality between the three different resuscitation regimens, all animals that died were replaced to maintain n=6 animals per group; there were no deaths among sham operated animals.

## Fluid infusion and hemodynamic response

**Figure 2:**
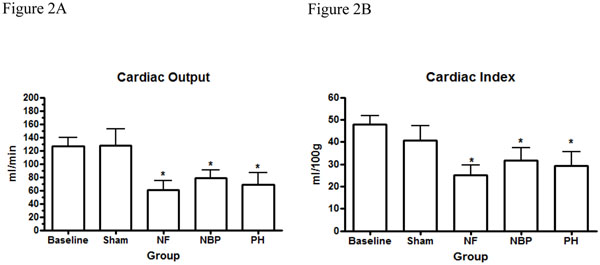
**Cardiac performance and resuscitation strategy.** Cardiac Output (Figure 2A) and Cardiac Index (Figure 2B) after hemorrhage and resuscitation. * *p* < 0.05 NF, NBP, and PH vs. baseline and sham groups; no statistically significant difference between NBP vs. PH (*p* > 0.05). NF = No Fluid; NBP = Normal Blood Pressure; PH = Permissive Hypotension.

**Figure 3:**
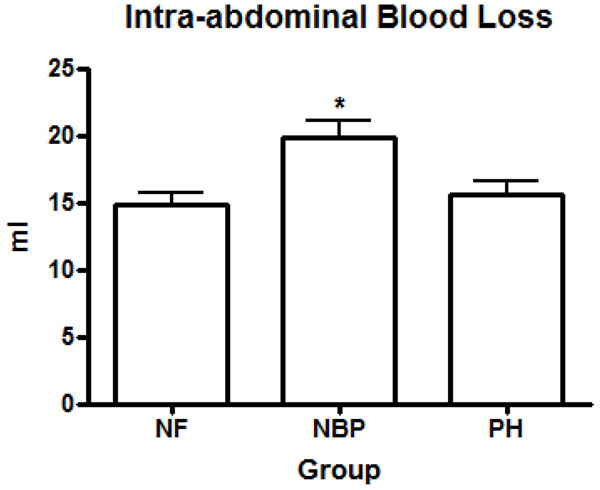
**Intraabdominal blood loss.** * *p* < 0.05 NBP vs. all other groups. NF = No Fluid; NBP = Normal Blood Pressure; PH = Permissive Hypotension.

Normotensive resuscitated animals received significantly more intravenous LR during resuscitation than PH animals (7.21 ± 3.24 ml/100g vs. 2.45 ± 1.05 ml/100g; *p* < 0.0001). Fluid infusion in sham operated animals and NF group were negligible. Baseline MAP were similar among the animals; average 92.6 ± 5.8 mmHg (*p* > 0.05). Aortic injury lead to uncontrolled bleeding and a significant reduction in MAP by 5 minutes in all hemorrhage groups compared to baseline levels and sham operated animals (Figure 1). The MAP in the normotensive resuscitated animals (NBP group) was successfully restored to baseline and sham operated animals in approximately 30 minutes after the beginning of the bleeding (71.9 ± 5.2 mmHg; *p* > 0.05). However, the MAP in the NF group and PH resuscitated animals remained significantly lower than NBP and sham groups, as well as baseline, until the end of the experiment (54.3 ± 1.5 mmHg and 61.1± 1.2 mmHg; *p* < 0.0001) respectively (Figure 1). The cardiac output and the cardiac index reduced significantly in all hemorrhage groups compared to baseline levels and sham operated animals. However, there was no statistical difference between the hemorrhage groups and the resuscitation regimen used (Figures 2A and 2B). Normotensive resuscitated animals (NBP group) presented significantly higher intra-abdominal blood loss (18.8 ± 3.5 ml/Kg) compared to the NF group (14.9 ± 3.2 ml/Kg), and the PH group (16.2 ± 3.9 ml/Kg); *p* < 0.05 (Figure 3).

## Organ perfusion

**Figure 4:**
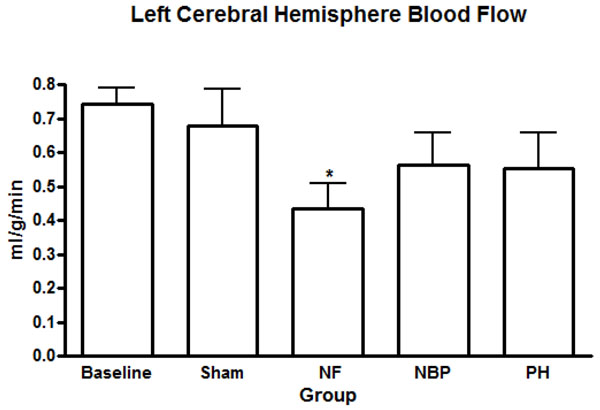
**Perfusion of the left cerebral hemisphere.** * *p* < 0.05 NF vs. baseline and sham groups; no statistically significant difference between NBP vs. PH (*p* > 0.05). NF = No Fluid; NBP = Normal Blood Pressure; PH = Permissive Hypotension.

**Figure 5:**
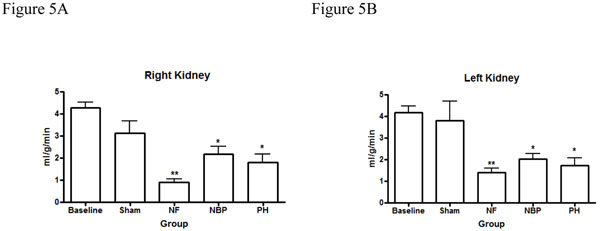
**Perfusion of the kidneys.** Right kidney (Figure 5A) and left kidney (Figure 5B), * *p* < 0.05 NBP and PH vs. baseline; ** *p* < 0.05 NF vs. all other groups; no statistically significant difference between NBP vs. PH (*p* > 0.05). NF = No Fluid; NBP = Normal Blood Pressure; PH = Permissive Hypotension.

**Figure 6:**
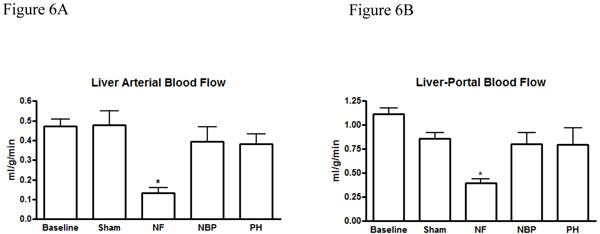
**Perfusion of the liver.** Arterial perfusion to the liver (Figure 6A) and portal perfusion of the liver (Figure 6B). * *p* < 0.05 NF vs. all other groups; no statistically significant difference between NBP vs. PH (*p* > 0.05). NF = No Fluid; NBP = Normal Blood Pressure; PH = Permissive Hypotension.

**Figure 7:**
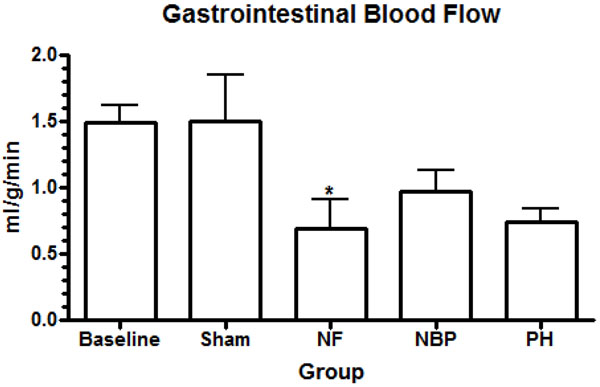
**Gastrointestinal perfusion.** * *p* < 0.05 NF vs. baseline and sham; no statistically significant difference between NBP vs. PH (*p* > 0.05). NF = No Fluid; NBP = Normal Blood Pressure; PH = Permissive Hypotension.

**Figure 8:**
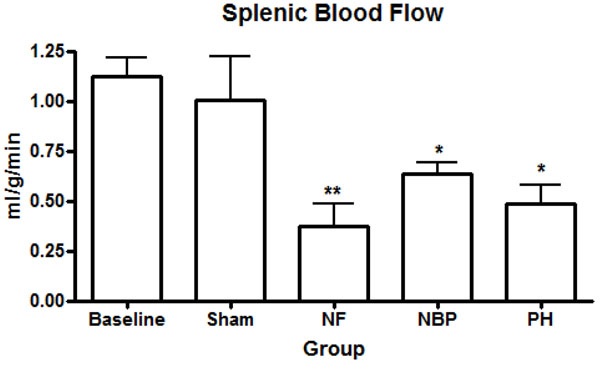
**Perfusion of the spleen.** * *p* < 0.05 NBP and PH vs. baseline; ** *p* < 0.05 NF vs. all other groups, no statistically significant difference between NBP vs. PH (*p* > 0.05). NF = No Fluid; NBP = Normal Blood Pressure; PH = Permissive Hypotension.

**Figure 9:**
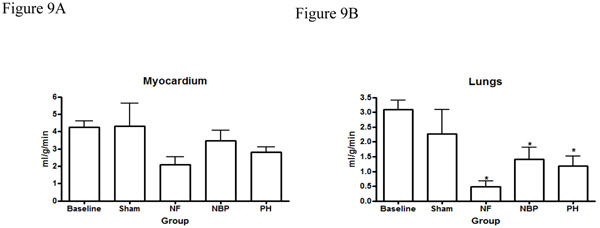
**Perfusion of the myocardium and lung.** Myocardial perfusion (Figure 9A) and lung perfusion (Figure 9B) after hemorrhage and resuscitation. * *p* < 0.05 NF, NBP, and PH vs. baseline and sham groups; no statistically significant difference between NBP vs. PH (*p* > 0.05). NF = No Fluid; NBP = Normal Blood Pressure; PH = Permissive Hypotension.

Our results showed no statistical difference in the cerebral blood flow between NBP and PH groups. However, cerebral perfusion reduced significantly in the NF group compared to baseline and sham operated animals (Figure 4). Renal blood flow reduced significantly in both kidneys after hemorrhage compared to baseline levels, NF group reduced renal blood flow, in both kidneys, compared to all other groups (Figures 5A and 5B). Arterial blood flow to the liver was significantly reduced in the NF group compared to all other groups (Figure 6A). The portal blood flow to the liver was also significantly reduced in the NF group compared to baseline levels; there were no statistical differences amongst the other groups (Figure 6B). The NF group showed a significant reduction in the gastrointestinal blood flow compared to baseline and sham operated animals; there was no statistical difference between NBP and PH groups (Figure 7). Blood flow to the spleen reduced significantly in the NF group compared to all other groups (Figure 8). However, splenic blood flow in the NBP and PH groups were only statistically different compared to baseline (Figure 8). No statistical difference was noted in the blood flow to the myocardium (Figure 9A). Blood flow to the lungs reduced significantly in all hemorrhage groups compared to baseline levels, regardless to the resuscitation regimen used (Figure 9B).

## Laboratory tests: hemoglobin, hematocrit, platelets, and serum lactic acid

**Figure 10:**
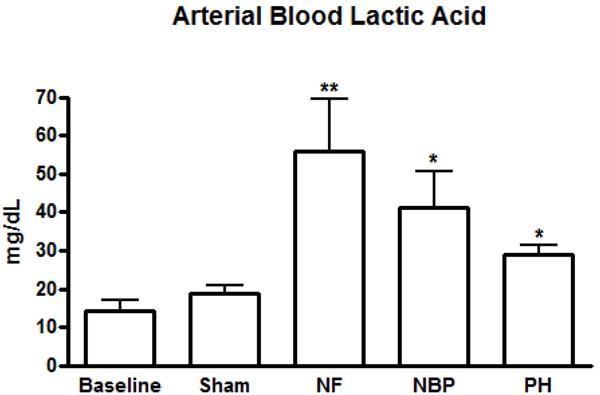
**Lactic acid levels in arterial blood.** * *p* < 0.05 NBP and PH vs. baseline and sham groups; ** *p* < 0.05 NF vs. all other groups; no statistically significant difference between NBP vs. PH (*p* > 0.05). NF = No Fluid; NBP = Normal Blood Pressure; PH = Permissive Hypotension.

All animals had similar baseline hematocrit, hemoglobin and platelet levels. A significant decrease in the hemoglobin and hematocrit levels occurred in all hemorrhage groups compared to baseline and sham operated animals (Table 1). The NBP group showed the lowest hematocrit and hemoglobin levels after hemorrhage (24.9 ± 4.0% and 9.0 ± 1.1 g/dL), respectively. Additionally, that group had significantly lower Hb and Hct levels than the NF group (Table 1); platelet count in NBP and PH groups reduced significantly compared to baseline. Lactic acid in the arterial blood was statistically higher in the NF group (55.9 ± 35.8 mg/dL) compared to all other groups. There was no statistical difference between NBP and PH groups lactic acid levels, although both groups showed higher levels than baseline and sham operated animals (Figure 10).

**Table 1:** Laboratory test results

	Baseline	Sham	NF	NBP	PH
Test					
Hct (%)	41.5 ± 3.4	32.7 ± 2.9	30.8 ± 3.0^*^	24.9 ± 4.0^*‡†^	28.5 ± 4.1^*‡^
Hb (g/dL)	15.0 ± 1.4	13.5 ± 1.0	10.8 ± 1.0^*‡^	9.0 ± 1.1^*‡†^	10.2 ± 1.2^*‡^
Platelet x 10^3^	623 ± 111	546 ± 87	993 ± 157	447 ± 185^*^	419 ± 71^*^

Hct, Hematocrit; Hb, Hemoglobin; NF, No Fluid; NBP, Nominal Blood Pressure; PH, Permissive Hypotension.

Data reported as mean ± SD.

* *p* < 0.05 vs. Baseline

‡ *p* < 0.05 vs. Sham

† p < 0.05 vs. NF

## Discussion

Permissive hypotension was described by Canon et al. as a resuscitation strategy in the acute phase of traumatic hemorrhagic shock more than 90 years ago [26]. The advantages of hypotensive resuscitation in the management of trauma related hemorrhage have been shown by several investigators in both experimental and clinical studies [3,6,7,9-13]. Current guidelines for trauma life support prudently indicate cautious fluid infusion in penetrating torso trauma until hemorrhage is controlled [3,4,6]. Accordingly, the present study showed that PH decreases blood loss compared to normotensive resuscitation. Furthermore, and more importantly, we showed that PH resuscitation did not reduce organ perfusion compared to NBP resuscitation after uncontrolled bleeding.

Concerns about organ hypoperfusion provoked by hypotensive resuscitation has been emphasized by several investigators [9,14,16-19,27,28]. Decreased organ perfusion causes oxygen debt that leads to intracellular hypoxia and damage to the mitochondrial membrane, resulting in the generation of free electrons and oxidative tissue injury [29-31]. Moreover, prolonged hypoperfusion provokes exacerbated inflammatory response involving a complex interaction of several pro and anti-inflammatory mediators, neutrophil activation, and specific patterns of cellular gene expression that can ultimately lead to multiple organ failure [32,33].

Even though PH resuscitation raises concern about organ hypoperfusion, several studies have shown that an overzealous fluid infusion strategy to prevent that complication is certainly harmful [34,35]. Large volume resuscitation provokes generalized increase in interstitial fluid and cellular edema that have been linked to organ dysfunction [34]. It was demonstrated clinically that supranormal resuscitation in major trauma patients, led to increased LR infusion and a higher incidence of abdominal compartment syndrome and multiple organ failure [35]. Excessive LR infusion, particularly the D-isomer of lactate, has also been implicated in increased expression of inflammatory genes and neutrophil adhesion molecules, as well as, in the stimulation of neutrophil oxidative burst [36,37]. Furthermore, excessive fluid infusion has been considered a major cause of coagulopathy in the acute hemostatic derangement of trauma patients recently termed Acute Coagulopathy of Trauma-Shock (ACoTS) [38]. Therefore, a resuscitation strategy concurrently involving judicious fluid infusion and adequate organ perfusion would be particularly beneficial in the management of the bleeding trauma patient [1,3-8,38].

Regional organ perfusion can be estimated experimentally by the microsphere deposition method. It was initially described in 1967 with radioactive microspheres, and has been validated by several investigators [24,25,39]. Because of legislation requirements, higher costs, and special care for the disposal and manipulation of radioactive material, non-radioactive microspheres were developed [21-24]. The fluorescent microspheres technique was introduced in 1993 and several studies showed comparable accuracy between fluorescent microspheres and radioactive microspheres in the assessment of systemic blood flow and organ perfusion [24,40-42].

In the present study the organs of the animals that underwent PH resuscitation showed equivalent fluorescence compared to normotensive resuscitated animals, suggesting similar organ perfusion but less bleeding. To verify the accuracy of our methodology we tested the perfusions of the left and the right kidneys before hemorrhage. A difference greater than 15% in the blood flow between the two kidneys suggests inadequate mixing of the microspheres and interferes with the accuracy of organ perfusion assessment [40,42]. Our results showed practically the same perfusion in both organs confirming adequate mixing of the microspheres in the left ventricle, thereby validating the process [40,42]. Perfusions of the brain and the myocardium were sustained during acute hemorrhage. Studies show that the cerebral vascular resistance decreases during hemorrhagic shock, temporarily maintaining cerebral blood flow within normal limits; a similar mechanism works in the myocardium [43,44]. We also speculate that the ligation of the right carotid artery, before the bleeding, may have augmented the compensatory mechanism in cerebral blood flow during hemorrhage in the NBP and PH groups.

As expected, we showed a decrease in CO and CI in all hemorrhage groups compared to baseline levels and sham operated animals, no statistical difference was detected between hemorrhage groups. Although that finding could be attributed to a temporary compensatory response of the cardiovascular system, Smail et al. report transient increased cardiac output in resuscitated animals compared to no resuscitation using radioactive microspheres 1.5 hours after the completion of resuscitation [25]. They also showed that increasing the resuscitation volume did not result in improved hemodynamics or organ perfusion [25]. Our results support that finding by the absence of significant difference in lactic acid levels in PH resuscitated animals compared to NBP resuscitation. However, we also demonstrated that a no fluid resuscitation strategy provokes significant organ hypoperfusion and increased lactic acid levels which is a marker of tissue hypoxia and has been linked to poor outcome in shock [45,46]. Additionally, we speculate that re-bleeding, particularly after the 50^th^ minute, partially explains hypoperfusion in the NBP resuscitated animals where the rate of fluid infusion had to be increased to maintain blood pressure within the preset limit. The potential for re-bleeding during normotensive resuscitation has been described by others [47,48].

The hemorrhage model used in our study adequately simulates a penetrating trauma to the torso and a major vascular injury. By closing the abdomen immediately after the aortic puncture we restored the tamponade effect of the abdominal wall, and at the same time, maintained an uncontrolled hemorrhage. Furthermore, we attempted to reproduce the time intervals between injury and EMS notification up to emergency room times [47,49,50]. Therefore, we believe that our model is clinically relevant and can be used to investigate resuscitation strategies during the acute phase of hemorrhagic shock in an urban setting [2,3,5-8].

There are limitations to be considered in our study. Hemodynamic response obtained from larger animals reproduces human physiologic derangement provoked by hemorrhagic shock more efficiently than from small animals. Another limitation of small animal models is the tendency for microspheres to deposit preferentially in regions of higher than average blood flow, thus creating potential error in the assessment of the perfusion to the heart and the brain [42]. However, such bias is reduced when microspheres in the range of 10 to 15 μm are used [42]. Dye loss from microspheres can also interfere with the accuracy of the method. However, dye loss is less than 1% with the methodology used in this study. Nonetheless, we assessed that potential limitation by randomly verifying the fluorescence of the supernatant of our samples and constantly obtained negligible results. Lastly, the total time of our experiment was set to simulate only the timing of events that take place acutely in trauma; until hemorrhage is definitively controlled. Therefore, any late and deleterious effect resulting from the three resuscitation strategies were not assessed in this study.

In summary, hypotensive resuscitation causes less intra-abdominal bleeding than normotensive resuscitation and concurrently maintains equivalent organ perfusion. No fluid resuscitation reduces intra-abdominal bleeding but also significantly reduces organ perfusion.

## Competing interests

The authors have no competing interests to disclose regarding the study.

## Authors’ contributions

JBRN conceived the study, participated in its design and coordination, drafted the manuscript and performed statistical analysis. BMS participated in the study design, carried out the assays, participated in the hemorrhagic shock procedures, helped to draft the manuscript and to perform statistical analysis. MVA participated in the study design and coordination. PCW carried out the assays and participated in the hemorrhagic shock procedures. MGC carried out the assays and participated in the hemorrhagic shock procedures. TAL carried out the assays and participated in the hemorrhagic shock procedures. SBR participated in the design and coordination, helped draft the manuscript. JRCM participated in the design, coordination and helped to perform the assays of the study. All authors read and approved the final manuscript.
